# Suicidal behavior among older adults: Effects on adult children’s mental health and family relationships

**DOI:** 10.1192/j.eurpsy.2026.10674

**Published:** 2026-07-31

**Authors:** S. von Humboldt, G. Low, I. Leal

**Affiliations:** 1 William James Center for Research, ISPA – Instituto Universitário, Lisbon, Portugal; 2 Faculty of Nursing, International Health Research, MacEwan University, Edmonton, AB, Canada

## Abstract

**Introduction:**

Suicidal behavior in later life extends beyond the individual, significantly impacting family dynamics and the psychological well-being of adult children.

**Objectives:**

This study aims to: (1) examine the effects of older adults’ suicidal behavior on their relationships with adult children; and (2) investigate the mental health consequences for adult children navigating these experiences.

**Methods:**

A total of 121 adult children whose older parents demonstrated suicidal behavior participated in this qualitative study. Thematic insights were derived from in-depth interviews through content analysis.

**Results:**

For the first objective, the findings showed five themes: Erosion of trust (81.8%); Fragile relationship dynamics (71.1%); Expanded caregiving duties (67.8%); Emotional disconnection (57.9%); and Reversal of family roles (54.5%). For the second objective, five themes emerged from the content analysis: Emotional overwhelm (80.2%); Persistent hypervigilance (76.0%); Depressive symptoms (63.6%); Post-traumatic distress (56.2%); and Emergence of suicidal ideation (34.7%).

**Conclusions:**

Suicidal behavior among older adults profoundly destabilizes both their emotional well-being and the relational bonds with their adult children. This study highlights the urgent need to extend support beyond the older individuals themselves to include their adult children. Effective interventions should address the distinct challenges these adult children face, offering tailored resources to reduce their emotional strain and promote mental health resilience. In addressing suicidal behavior within families, it is essential to acknowledge the often-invisible sacrifices made by those who carry the weight of their loved one’s crisis.

Keywords: Suicidal behavior; older adults; adult children; mental health; qualitative studies; intergenerational relationships.

**Disclosure of Interest:**

None Declared

